# Observing positive and negative social touch triggers topographically distinct physical and emotional reactions in the body

**DOI:** 10.1038/s41598-026-64569-7

**Published:** 2026-08-01

**Authors:** Justyna Świdrak, Wenhan Sun, Pinar Ekin, Merle T. Fairhurst

**Affiliations:** 1 Institute of Psychology, Polish Academy of Sciences (IP PAS), Warsaw, Poland; 2 Systems Neuroscience, Institut d’Investigacions Biomèdiques August Pi I Sunyer (IDIBAPS), Barcelona, Spain; 3 Centre for Tactile Internet with Human-in-the-Loop (CeTI), Faculty of Electrical and Computer Engineering, Technische Universität Dresden, Dresden, Germany

**Keywords:** Affective reactions, Body mapping, Interpersonal touch, Body representation, Chronic pain, Neuroscience, Psychology, Psychology

## Abstract

Interpersonal touch is a fundamentally multidimensional and multisensory experience and as such is highly subjective. By whom, where and how we are touched determines how we react to and feel about it. Previous research using topographical body tasks has mainly examined where people feel comfortable being touched by others. Using the BodyMap Tool, we mapped distinct somatoperceptions associated with positive and negative social touch videos, comparing physical sensations and emotional responses. We quantified these patterns with a BodyMap Score (BMS) reflecting valence and spatial coverage. Positive videos elicited higher physical than emotional BMS, whereas negative videos showed the opposite pattern, with broader emotional than physical somatoperceptions. Individual differences further modulated these effects: touch-avoidant participants showed reduced BMS for positive touch, and participants reporting pain exhibited overall lower BMS, though this reduction was attenuated for positive stimuli. These findings highlight the complex and context-dependent bodily representation of social touch.

**Supplementary Information:**

The online version contains supplementary material available at 10.1038/s41598-026-64569-7.

## Introduction

The same hug can feel warm and cosy or awkward and paralysing, even though its objective characteristics, such as touched area, force or duration, remain the same. This is because affective social touch is fundamentally a multidimensional^1^ and multisensory experience^2^. By whom, where, when, and how touch is initiated gives it meaning and heavily influences how we react to and feel about it ^3^. These context-dependent and often visually cued responses have observable manifestations on the body, forming reliable and topographically distinct somatoperceptions, or body maps that support embodied and culturally universal views of affect^4,5^. Somatoperception refers to the process of perceiving the body itself, and particularly of ensuring somatic perceptual constancy, and includes the localisations of the somatic stimuli on the body and the affective processing of and responses to somatic stimuli^6^. In this paper, we will discuss topographical somatoperceptions of vicarious social touch, using the term “body maps”. The study is also embedded in a broader embodied emotion framework, which proposes that conceptual processing of emotion is grounded in somatosensory and interoceptive systems^7–9^.

It is important to begin with a clarification of terms and their meanings, as various terms can be found in the literature. Previous research on so-called “bodily sensations” has tried to capture the impact that a stimulus has on a participant’s body. These stimuli range from words, stories, facial expressions and musical excerpts, and they elicit subjective bodily representations even though no direct bodily stimulation is present. In particular, Putkinen and colleagues^10^ demonstrated that bodily sensations contribute to the elicitation and differentiation of music-induced emotions, and suggest a similar embodiment of music-induced emotions in geographically distant cultures^10^. Others have used the same emBODY Tool^4^ and the term of bodily sensations to explore pain, tactile and hedonic sensitivity as well as emotional state in chronic pain patients^11^. However, we would propose that the term “sensation” may be misleading and would instead limit its use to tap into responses to physical events occurring to or on the body. These and other studies typically make reference to a “representation” (e.g. *bodily representation*^11^; *bodily representation*, *somatotopic representation* and *sensorimotor representation*^4^. We avoid the term *representations*, following the distinction made by Longo, Azañón, and Haggard^6^. The authors draw a clear distinction between *somatoperceptions* (low-level) and *somatorepresentations* (high level), with the latter defined as emotional states and attitudes *about* the body, best described as representations of one’s own body, and attitudes towards one’s own body that have emotional significance. Instead, we talk about *somatoperceptions*, defined by them as somatoperceptual processes related to experienced emotions in the body.

Adding to a growing body of work exploring vicarious touch events^12–14^, here we explore how the mere observation of positive and negative touch events results in either a physical or emotional response, and test participants’ ability to map distinct so-called *somatoperceptions*. To capture more generally the idea of tactile somatoperception, that is, the location and intensity of somatoperceptual responses to touch events (either physical or vicarious), we use the BodyMap tool and explicitly ask participants to report on where and how intensely they felt these on or in their body.

Different body maps coexist in parallel, for example, for experiencing pain, emotions, and touch, which implies multiple, partially overlapping codes for affective and sensory states^11,15^. For example, physical exercise triggers various, even contradictory, bodily reactions, such as exhaustion, activation and relaxation, all at once in distinct body areas^16^. The experience of pain also involves more than one component, including both sensory and affective dimensions^17,18^, each linked to a separate neural network^19^. For example, Ojala and colleagues^11^ demonstrate that bodily experience of emotion and pain as well as self-reported nociceptive, tactile, and hedonic sensitivity differ across chronic pain and matched healthy controls. In particular, people with chronic pain marked larger, condition-consistent localised nociceptive sensitivity while also exhibiting globally dampened embodied emotion maps. Similarly and intuitively, we know that a handshake may evoke a physical perception on the hand and an emotional perception of awkwardness in other areas of the body due to the handshake’s context. However, to date these different topological maps have not been explored for touch. The social-affective meaning of touch is encoded on the body and social touch shows relationship-specific bodily patterns, for example, more “touchable” (larger allowable touch) areas for closer relationships across cultures^20^. Furthermore, individuals with body image disturbances preferentially rate imagined loved-one touch as more soothing and to socially acceptable regions, highlighting how social context and bodily maps intersect^21^. More recently, we have shown that body maps detail topographical selectivity for social touch as a function of threat sensitivity^22^. However, despite the implied affective value of social touch, to date, there are no systematic comparisons of the physical compared with the emotional bodily responses to positive and negative touch events. Here, we apply the circumplex model of affect to explore the physical and emotional embodied responses to vicarious touch events. While both positive and negative touch events may share similar touch types and body parts touched^23^, how these touch events are felt will clearly vary as a function of context and meaning. Moreover, we posit that valence may modulate both localisation and intensity of these bodily feelings, as emotions are linked to distinct body maps. In particular, valence-related sensations of bodily lightness are linked with positive emotions (happiness, love, pride), while sensations of bodily heaviness are a response to negative emotions (e.g., anger, fear, sadness, depression), each with its specific body topography^24^.

How we experience touch also depends on individual differences. For example, attachment style and touch sensitivity/preferences systematically shape bodily experiences and emotions. People differ in affective touch sensitivity and desire for touch, influenced by behavioural inhibition, stress, gender, attachment, and relationship quality, which in turn biases how bodily affect is perceived and where it is permitted^25–27^. For instance, in a study on mother-infant touch, three subscales of the Multidimensional Assessment of Interoceptive Awareness Questionnaire (Attention regulation, Self-regulation and Body listening, see^28^ were significant mediators in the association between maternal tactile biography and maternal use of stroking in the relationship with her infant^29^. Additionally, Butti and colleagues^30^ found an interesting relationship between MAIA Trusting subscale and the evaluations of touch and the touching person. In particular, greater trust towards one’s own body and a stronger feeling that it is a safe placewere associated with higher appraisals of the touch pleasantness for another person, and for the person delivering the touch^30^. The Social Touch Questionnaire (STQ), an index of social touch avoidance/aversion, on the other hand, successfully distinguished between participants with low and high social anxiety, where those with high levels of anxiety (compared to low) rated social touch as more unpleasant and avoided it more across a variety of social situations^31^. We posit that both our individual preferences for touch and our degree of bodily awareness will determine the physical and emotional impact of touch events.

The current study evaluates the potential differences in topographical tactile somatoperceptions (operationalised here as body maps) between pleasant and unpleasant vicarious touch events and the affective reaction to it. Additionally, we test the influence of individual traits, touch aversion and interoceptive awareness, on the extent and intensity of these bodily feelings. Exploratively, we also ask whether people with chronic pain are characterised by either physical or emotional experiences of valenced touch events differently in terms of both location and perceived valence compared to those with no pain. We have previously employed the BodyMap tool to capture a BodyMap Score (BMS)^21^ as a measure of the size and valence of body accessibility or touchability. Here for the first time, this type of tool is used to characterise the multidimensional nature of social touch extending previous research which typically describes the intention or content of a social touch event as *affective*, to instead consider the differences between the physical and affective bodily responses to these events.

## Results

### Topological results

**Fig. 1:**
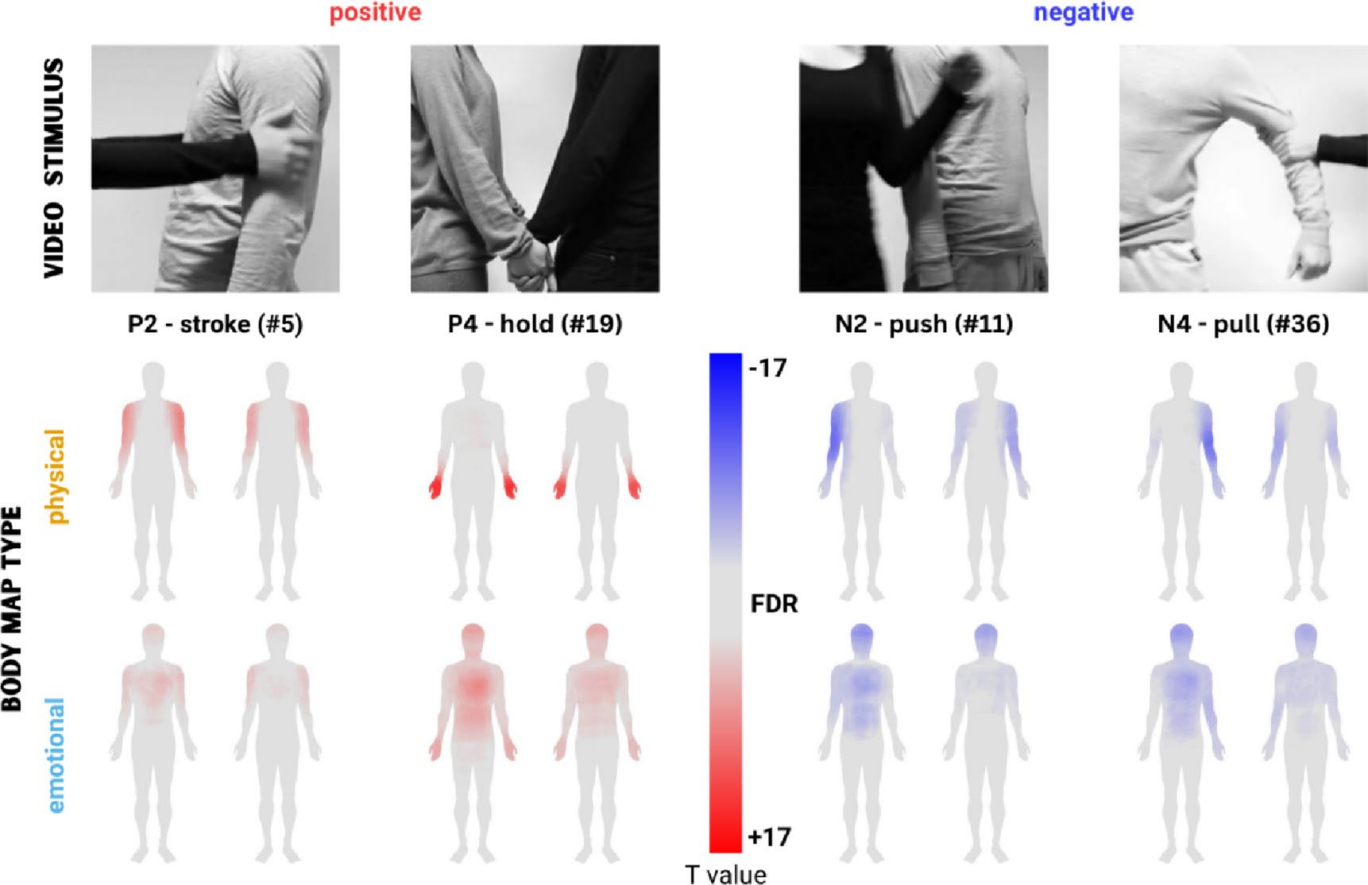
T-maps of the physical and emotional body maps of somatoperceptions evoked by each video. Results were obtained from univariate pixel-wise one-sample t-tests with FDR correction at α = 0.05. The colour bar indicates the range of t-statistics. Numbers (#) refer to the original numbering in the Socio-Affective Touch Expression Database (Table 1 in ref.^31^).

We find significant differences between the physical and emotional body maps (see Appendix Figure S2). For example, a video depicting handholding triggered a clearly defined physical reaction on the participant’s hands and a comparatively weaker (lower intensity of heatmap) emotional reaction in their chest, head, and hands (Fig. 1, left). By contrast, a negative video, for example one which presented an abrupt pushing, resulted in a physical body map with a strong lateral reaction on the right arm, corresponding to the right arm of the actor in the video, while the related emotional reaction was not experienced in the arm at all. Instead, it spilled over the head and torso (Fig. 1, right).

### BodyMap Score

#### BodyMap type and video valence

**Fig. 2:**
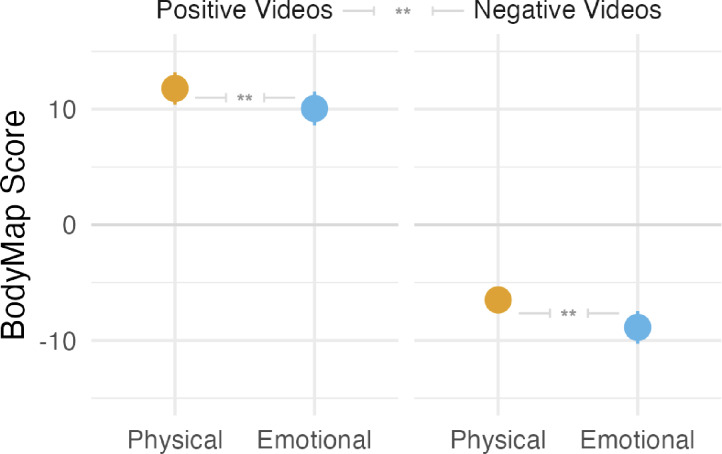
BodyMap Score for physical and emotional body map type as a function of video valence. Points indicate means; vertical lines show 95% confidence intervals (mean ± 1.96 SE). Significance stars reflect results from linear mixed effect regression (see Appendix Table S3 for more details).

We first examined how BMS is influenced by the BodyMap type and video valence using a mixed-effects linear regression, with random intercepts for participant and video (Appendix Table S3). We found that overall the emotional body maps exhibited a lower BMS (β=-2.087, SE = 0.657, *p* < 0.01) compared to the physical body maps. In addition, positive videos showed a substantially higher BMS (β = 18.567, SE = 4.205, *p* < 0.01) than negative videos. It should be noted that BMS is a bipolar continuous measure from positive to negative. When interpreted separately by direction, the results indicate that physical BMSs are generally more positive than emotional body maps (see Appendix Table S1 for more details; “hand holding” is one exception where we see the reverse trend) in positive videos. In contrast, emotional body maps are more negative for negative videos, compared to the physical manifestation of the same event (Fig. 2).

#### Subjective arousal and subjective valence

Next, we examined how BMS is influenced by subjective arousal and subjective valence using a mixed-effects linear regression, with random intercepts for participant and video (Appendix Table S4).

**Fig. 3:**
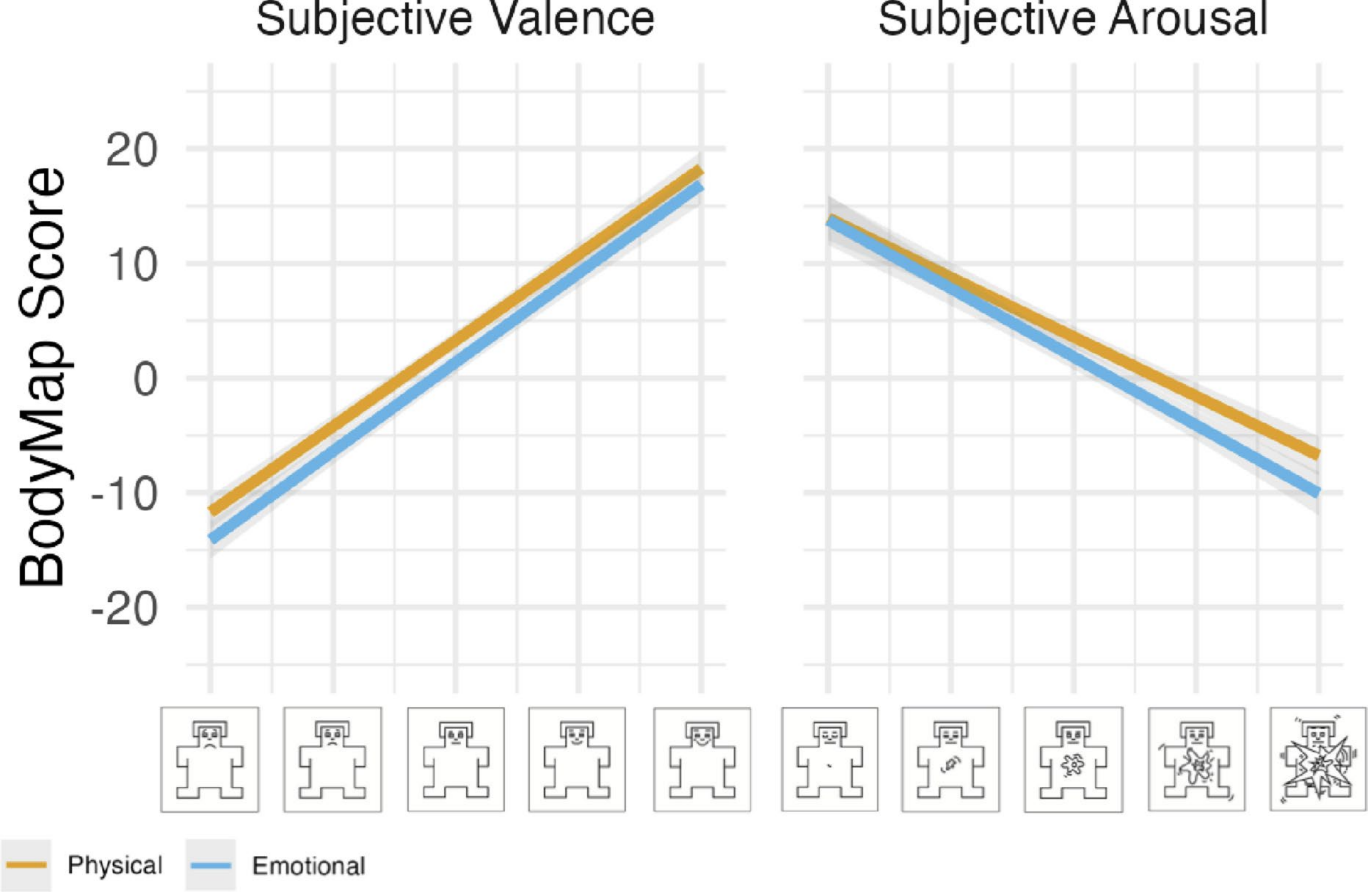
Relationship between subjective ratings of valence and arousal (SAM) and BodyMap Score. Lines are linear regression fits with shaded 95% confidence bands, coloured by Body Map Type (physical vs. emotional). Significance stars reflect results from linear mixed effect regression (see Appendix Table S4 for more details).

Subjective arousal was associated with a decrease in BodyMap Score (β=-0.837, SE = 0.150, *p* < 0.001). In contrast, subjective valence was associated with an increase in BMS (β = 2.669, SE = 0.195, *p* < 0.001), and the magnitude of this effect was substantially larger than that of arousal. Again, interpreting BMSs separately by positive and negative directions, we see more negative emotional body maps compared to the physical maps, for stimuli rated as low valence (“unhappy”, i.e. more negative videos). Additionally, for high arousing videos (“stimulated”, i.e. potentially more salient), we see more negative emotional BodyMap Scores. At the opposite ends of the subjective arousal and valence scales, the physical and emotional maps are more comparable (Fig. 3).

#### Individual traits and BodyMap Score

Finally, we investigated how BMS is influenced by individual traits using a mixed-effects linear regression, with BMS with BodyMap type, video valence, STQ score, MAIA score, pain status, and gender as fixed effects, with interaction terms between video valence and STQ, video valence and MAIA score, and video valence and pain status, and with random intercepts for participant and video (Appendix Table S4).

**Fig. 4:**
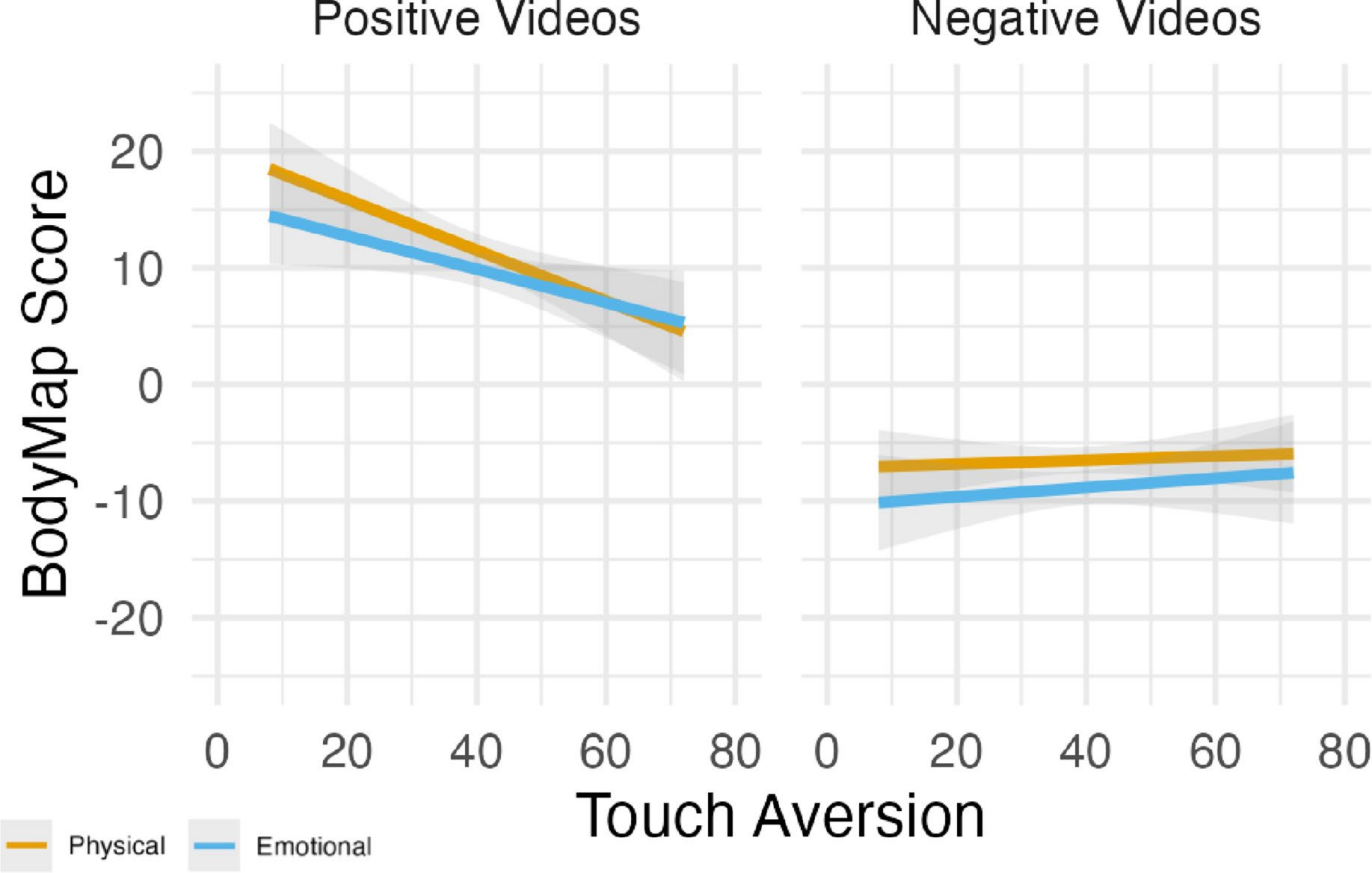
Relationships between social touch avoidance (STQ: Social Touch Questionnaire scores) and BodyMap Score, shown separately for positively and negatively valenced videos. Lines represent linear regression fits with shaded 95% confidence bands, coloured by Body Map Type (physical vs. emotional). Significance stars reflect results from linear mixed effect regression (see Appendix Table S5 for more details).

The main effect of STQ was not significant (β = 0.041, SE = 0.045, *p* = 0.366), however there was a significant interaction between STQ and video valence (β=-0.212, SE = 0.056, *p* < 0.001)(Fig. 4). This indicates that the relationship between STQ and BMS differed across valence conditions: while STQ showed little association with BMS for negatively valenced videos, higher STQ scores were associated with lower BMS for positively valenced videos (β=-0.171).

**Fig. 5:**
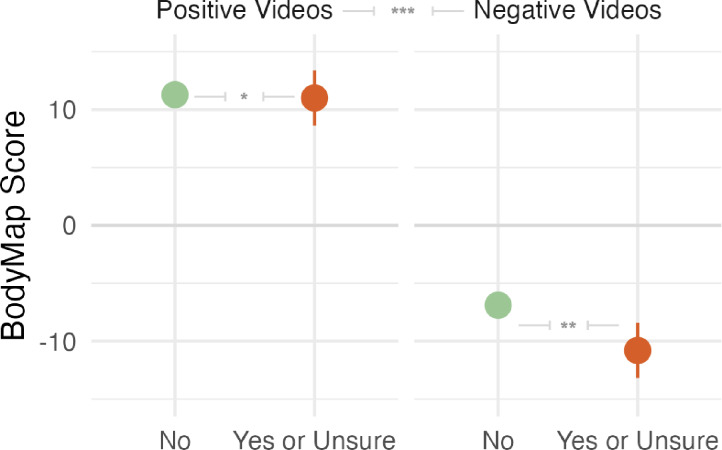
BodyMap Score and reported pain status (No vs. Yes or Unsure), separately for positive and negative videos (facets). Points represent group means, and vertical lines indicate 95% confidence intervals (mean ± 1.96 SE). Significance stars reflect results from linear mixed effect regression (see Appendix Table S5 for more details).

Pain status had a significant main effect on BMS. Participants who reported having or being unsure about having chronic pain had lower BMS (β=-3.688, SE = 1.265, *p* < 0.01) than those without pain. This was qualified by a significant interaction with video valence (β = 3.116, SE = 1.576, *p* < 0.05), indicating that the reduction in BMS associated with pain was diminished for positively valenced videos (β=-0.572) (Fig. 5).

Neither the main effect of MAIA nor its interaction with video valence was statistically significant, suggesting interoceptive awareness may play a limited role on BMS. Male participants have slightly higher BMS (β = 1.882, SE = 0.845, *p* < 0.05), compared to female participants.

## Discussion

The main objective of this study was to test whether vicarious touch evokes topographically distinct tactile somatoperceptions, depending on whether participants were asked to report on the physical or the emotional response to the viewed touch events. To do that, we asked participants to watch positive and negative social touch videos and to paint on a body silhouette to indicate where they felt it, first physically and then emotionally. Based on this information, we calculated the BodyMap score, which mathematically reflects the topographical responses. We posit that topographical perceptions of vicarious touch events are significantly predicted by the touch event, body map (physical or emotional), and impacted by individual differences. For positive videos, physical BodyMap scores were more positive than emotional ones. The reverse pattern was observed for negative videos, where emotional responses showed more negative effects than physical sensations. Subjective ratings of valence also positively predicted higher BodyMap Score, while subjective arousal predicted it in the opposite direction. We also found an interaction between the video valence and individual differences, with people more averse to social touch reporting higher BodyMap scores for positive videos and people in pain scoring lower on BodyMap score for negative videos. Lastly, we found small gender differences, with men having an overall higher BodyMap Score than women.

### Social touch as a multisensory and multidimensional experience

Hedger et al. have recently detailed the neural intertwining between our visual and tactile experiences of the world, showing that simply seeing films of certain body parts results in somatotopic tuning of the visual cortex^14^. They suggest that this two-way interaction between the visual and tactile systems allows for vision to inform touch and vice versa. We extend this by providing further evidence of the multisensory nature of social touch^2^. Until now, characterisation of the affective nature of social touch has primarily focused on describing the overall valence of a social touch event (e.g., pleasantness) without exploring its embodied nature (see^32^ for a comparison of physical and emotional qualities of CT-mediated touch to three body sites). Separately, descriptions of the physical nature of these events have generally focused on detailing how low-level sensory parameters (e.g., velocity^33^ alter perception at a limited number of body sites^34^. Here, instead, we have agnostically prompted participants to consider their whole body and to map how they feel about vicarious touch events and demonstrated that it is possible to maintain and report on two separable and distinguishable body perceptions related to a single vicarious touch event^1,35^.

Looking further at the multidimensional nature of body maps, we can refer to the circumplex model of affect, which states that valence, a cognitive interpretation of a situation, determines the direction (approach vs. avoidance) and arousal determines the intensity of basic affective responses^36^. Based on our results, physical and emotional body maps may differentially relate to valence and arousal separately, or to some combination of these two dimensions (e.g., is the emotional map more heavily weighted towards arousal while remaining valenced). Specifically, that highly arousing and more negative videos modulated more strongly the emotional body maps supports the claim that emotions are not only generally, but also specifically and locally embodied.

### Beyond topographical descriptives

Topologically, we computed topographical maps which identify body regions that participants consistently rated as positive or negative^4,5^. This approach revealed clear visual differences across conditions and allowed for intuitive inference of systematic spatial patterns. In comparison to physical body maps, effects in emotional body maps often emerge in different regions, most notably the head and torso, and often span larger areas of the body. Mathematically, we derived a new BodyMap Score to quantify these effects in a compact representation of overall body map activation, which allowed for including this type of data in multivariate statistical models. Additionally, for exploratory purposes, we have calculated mean pixel activation restricted to painted/non-neutral regions (BodyMap Intensity) which primarily captures intensity, and the proportion of the body painted (BodyMap Coverage) to isolate spatial extent. We have included examples of this approach in the supplementary materials (see Appendix Figure S2, S3; Appendix Table S1). Although systematic comparisons of these metrics fall outside the scope of the present study, we feel that such an approach is an important direction for future research, which may more comprehensively account for differences between the physical and emotional body maps.

### Are emotional somatoperceptions really smaller?

Based on a continuous bipolar scale of BodyMap Score, we find a main effect of body map type, with emotional body maps showing overall lower BodyMap Scores. The magnitude of BMSs can be interpreted separately for the positive and negative direction. While for most positive events (except for hand holding), we observe greater positive intensity for physical maps compared to emotional responses. For negative touch events, the opposite is true, with emotional body maps having higher negative intensity than physical body maps. These results can be driven in part by differences in the spatial extent of painted areas (see Appendix Figure S6), where we see that BodyMap Coverage of bodily feelings is generally lower across both positive and negative videos, except the hug videos. However, this pattern may be because the negative touch events were relatively localised, with the poke, push, and pull touches targeting only one side of the body. Furthermore, participants in our study were not instructed to imagine anyone in particular in the role of the touching person, and this lack of a relational context could potentially dampen the emotional reaction to positive touch events^3,21,27^. Looking at subjective ratings, we found that the more negative and arousing/stressful the touch event, the more localised the tactile somatoperception.

### Head-heart-centred emotional somatoperceptions of affective touch

Physical sensation maps closely tracked the specific body regions touched in the videos, with lateralised responses when the touch was targeted to one side of the body and limited to where contact was made. By contrast, emotional body maps showed a markedly different organisation that aligns with prior work on affective bodily perceptions^10^. In particular, emotional responses were characterised by prominent head- and chest-centred hotspots. For example, Putkinen and colleagues^10^ showed that tender, sad, and scary musical stimuli reliably elicited increased activation in the head and heart regions, an effect that was less pronounced for happy stimuli. Interestingly, but conflicting with these findings, Hartmann, and colleagues find hot spots for “happy” emotions in the head and heart^24^. Saanijoki and Nummenmaa^16^ reported head-centred activation during calm and peaceful post-exercise states, while breathless conditions,characterised by heightened interoceptive load, were associated with pronounced chest activation, which they interpret as reflecting interoceptive awareness of autonomic changes, such as alterations in heart rate and respiration, noting that scary stimuli, despite being emotionally distinct, shared bodily signatures with sad and tender stimuli due to similarly low energisation and arousal.

Similarly, we observed head- and heart-centred emotional hotspots across both positive and negative touch events, suggesting a common autonomic arousal component rather than touch-specific somatosensory encoding. This interpretation is further supported by recent work linking social touch to modulation of autonomic and interoceptive processes^37–39^, reinforcing the view that emotional bodily maps primarily index arousal-related physiological states rather than the spatial locus of physical contact.

### Sensory / physical nature of touch events related to social touch avoidance

Our previous work has shown that preferences for social touch influence reported touchability^22^. In the present study, touch avoidance predicted differences in physical body maps, with individuals more receptive to social touch showing higher physical relative to emotional BodyMap Scores. Touch avoidance also interacted with touch valence: it had little effect for negative videos but was associated with lower scores for positive touch, suggesting that affectionate touch may be less embodied by touch-avoidant individuals. This is an interesting result, as it suggests that hugging and other forms of affectionate touch may not be felt on the body by touch-avoidant individuals. The causality of this relationship remains yet unknown, although the literature on touch aversion suggests several potentially relevant facts. For example, touch-avoiders characterise themselves as having low body awareness, described as the feeling of not knowing the physical limits of the body, and of being physically clumsy, which can be a consequence of not having been physically touched, that is embraced, to a sufficient degree^40^. Yet another research line has demonstrated that touch avoidance is associated with avoidant attachment style^26^, and more autistic traits^41^, and in autistic people, the attitude towards social touch represents a central disposition contributing to lower levels of social touch erogeneity, pleasantness, and appropriateness^42^.

### Presence of pain and body maps

In pain-free individuals, positive social touch is generally experienced as pleasant, whereas conditions such as chronic pain are associated with altered touch perception^43,44^. Body maps are therefore widely used in pain research to distinguish localised and centralised pain mechanisms^45^. In our study, participants with pain showed lower BodyMap Scores than pain-free participants, although this reduction was attenuated for positive videos. This is in line with Ojala et al. ^11^, who reported dampened body maps in pain patients despite preserved sensitivity to hedonic touch. Together, these findings suggest that chronic pain alters bodily experience and distorts how social touch is embodied, although due to the modest size of the pain subgroup, these results are of exploratory nature and require further, systematic studies on larger and more homogenous populations.

### Study limitations and future directions

A limitation of the present study, shared by the broader body-mapping approach, is that BodyMap responses may reflect a cognitively mediated evaluation of bodily experience rather than a purely spontaneous affective response. As with all subjective self-report measures, some degree of reflection is inherent. However, the BodyMap tool explicitly directs participants to focus on experienced changes in their bodies (“… colour the regions of your body where you physically sensed the touch while watching the video” and “colour the regions of your body that you felt changing emotionally while watching the video”) and does not require verbalisation of the experience. We therefore suggest that it may capture affective responses in a more embodied and less semantically constrained manner than conventional self-report measures, combining an initial bodily-affective experience with subsequent evaluation. Nevertheless, the extent to which BodyMap responses correspond to reactions elicited by actual touch remains an open empirical question, and reflective processes may differ between vicarious and directly experienced touch, particularly for salient or negatively valenced events.

This study presented touch stimuli without context to capture general responses, but social touch is inherently contextual. Future work should examine how relational and situational factors shape physical and emotional body maps, for example, by asking participants to imagine a specific person or motivation for the touch. The modest sample size limited analyses of demographic effects, highlighting the need for larger, more diverse samples. Additionally, future studies should investigate more homogeneous chronic pain groups to clarify how pain-related body disturbances influence social touch perception.

## Conclusions

This study demonstrates that vicarious social touch evokes distinct physical and emotional somatoperceptions that can be visualised as topographical body maps. Physical maps are spatially precise and closely tied to observed contact, whereas emotional maps are broader and centred on head- and chest-related interoceptive regions. Using the BodyMap tool, we show that these somatoperceptions are shaped by touch valence, subjective arousal, social touch avoidance, and chronic pain. Together, our findings highlight the complex, multidimensional nature of social touch and its embodied representation in the human body.

## Methods

### Participants

The study was conducted on the *Gorilla.sc* platform in July and August 2024. A power analysis was performed with Gpower 3.1 software. The power was calculated for a two-tailed *F* test to compare between the physical and emotional body maps in 12 videos, with a power 0.95, and an alpha level of 0.05. a minimal sample size of 100 was required for a small-to-medium-effect size (Cohen’s d = 0.4). Participants were recruited for the study through the Prolific (www.prolific.com) online research platform. Data were initially obtained from 154 participants. Written informed consent was obtained from all the participants. Six participants were excluded due to the absence of any painting responses across all videos in the paint task. Consequently, data from 148 participants (64 female and 84 male) were retained for all subsequent analyses. Participants ranged in age from 18 to 71 years (mean = 31.00 ± 10.70). Roughly half of the sample (*N* = 60, including 16 women) were single, while the rest were either in a formalised or informal relationship. Participants were located in all continents except Antarctica. Eighteen female and 16 male participants reported suffering from chronic pain or being unsure whether they experienced chronic pain, and three male participants preferred not to say. In most cases, it was musculoskeletal pain. The study was performed in accordance with the Declaration of Helsinki. All participants provided informed consent, and ethical approval was obtained through the Technical University of Dresden’s ethics committee (SR-EK-42012023). The research was performed in accordance with relevant guidelines and regulations.

### Materials

**Demographic questionnaire.** A short demographic form was created to collect information on participants’ gender, date of birth, nationality, education level, relationship status, occupation, and whether they experienced chronic pain. **Social Touch Questionnaire (STQ).** The Social Touch Questionnaire (STQ) is a self-report measure used to capture touch avoidance^22^. The questionnaire consists of 20 items, which participants rate on a scale from 1 (not at all) to 4 (extremely). **Multidimensional Assessment of Interoceptive Awareness (MAIA)** is a self-report tool developed by Mehling et al. (2012) to evaluate awareness of internal bodily signals^46^. This updated version comprises 37 items rated on a scale from 1 (never) to 5 (always) and is divided into eight subscales: Noticing, Not-Distracting, Not-Worrying, Attention Regulation, Emotional Awareness, Self-Regulation, Body-Listening, and Trusting.

We selected 12 out of 75 videos from **Socio-Affective Touch Expression Database**^47^, selected for their valence (positive, negative, neutral). In all selected videos, touch events targeted upper body regions. Two of them represented touching an object (lifting a water battle, shaking a small battle), one representing tapping of someone’s shoulder, and one pushing a door aggressively. The neutral videos were control videos, used for a sanity check. T-maps and descriptive data for them are reported in the Appendix, and they are excluded from further statistical models.

**Subjective valence and arousal** was measured with the Self-Assessment Manikin (SAM), a picture-based questionnaire, designed to assess affective reactions to stimuli^48^. After watching the video, participants were asked, *How did the video make you feel? Please*,* select one number in each row using the drawings as a scale.* Their task was to mark the manikin on a 9-point scale for each touch event. The poles of the two items were worded respectively “Unhappy” to “Happy” and “Relaxed” to “Stimulated” for increased clarity (Appendix, Figure S4).

A JavaScript-based **BodyMap Tool** (available at https://github.com/justinsulik/body_paint*)* was used to enable participants to paint on body silhouettes to indicate where and how they felt bodily sensations after watching each video. Both the front and back of the body were presented as body silhouettes. Participants could select different colours which indicate positive and negative experiences and their magnitudes, as well as sizes of paint brushes (Appendix, Figure S5).

### Procedure

The experiment was created and hosted using the Gorilla Experiment Builder platform (www.gorilla.sc*)*, and the study was conducted entirely online^49^. The total duration of the experiment was approximately 25 min. After providing informed consent, participants filled in the demographic form, and the completed STQ and MAIA questionnaires. Next, participants were informed that they would watch a series of videos depicting touch events and were instructed to pay attention to and later rate two specific aspects while viewing the videos: (1) where they physically felt the touch on their bodies and (2) how the touch made them feel emotionally. After viewing each video, participants completed the SAM items and the BodyMap task (Appendix, Figure S5). They used colour gradients ranging from red (positive affective change) to cyan (negative affective change) to indicate the valence and intensity of their perception For each video, participants coloured in two body maps (physical, emotion) with two silhouettes (front, back). Each participant viewed all 12 videos; each video can be played up to 3 times. The order of videos was fully randomised across participants.

### Data analysis

Paint-stroke data gathered from each body map task were processed using a Python script provided with the BodyMap tool, and rendered into images for further analysis. Subsequent data processing and statistical analyses were performed in R^50^. Each pixel was converted into a continuous pixel activation value based on its colour. The values range from − 100 (maximum negative) to + 100 (maximum positive), with neutral being 0.

To identify regions showing consistent bodily perceptions of touch across participants, we performed univariate pixel-wise one-sample t-tests (based on ^4,5^) on body maps for each video and for each body map type (physical, emotional), with false discovery rate (FDR) correction at α = 0.05. This procedure yielded thresholded statistical summary maps (t-maps), in which different colours indicate different levels of statistically significant bodily activations associated with each stimulus across participants.

To quantify the effects in each body map, we computed the **BodyMap Score** based on the mean pixel activation value on the entire body. This score reflects both the intensity and extent of painted/non-neutral feelings, and is used as the primary measure for statistical analysis.

We considered 2 alternative approaches of mathematisation. **BodyMap Intensity** score was computed based on the mean pixel activations in non-neutral pixels and reflects primarily the intensity of painted feelings. **BodyMap Coverage** was computed based on the ratio of painted pixels in the entire body and reflects the extent of painted feelings. Only descriptive statistics for these measures are reported.

Mixed-effects linear regressions (LMERs) were performed to examine the relationships between BodyMap Score and other measurements. The models included fixed effects of experimental conditions and stimulus-related measures: BodyMap type, video valence, subjective valence and subjective arousal; as well as individual difference variables: STQ score, MAIA score, pain status, and gender. For pain status, we clustered participants who responded “Yes” and “Unsure”, as the latter group most likely had experienced pain, although they may have not received an official “chronic pain” diagnosis or had doubted whether their pain classifies as chronic. It seemed unlikely that a person without pain would have selected the “Unsure” answer. The statistical baseline for the body map type (physical, emotional) was physical, for valence (negative, positive) was negative, and for gender (female, male, non-binary) was female. Random intercepts were included for participant and video to account for repeated measures within individuals and across stimuli.

## Supplementary Information

Below is the link to the electronic supplementary material.

Supplementary Material

## Data Availability

All experimental data supporting the findings of this study are available on the Open Science Framework (OSF) at https://osf.io/j4kea. Additional data may be requested from the corresponding author.

## References

[1] Lee Masson, H. et al. The multidimensional representational space of observed socio-affective touch experiences. *Neuroimage* **175**, 297–314 (2018). 29627588 10.1016/j.neuroimage.2018.04.007 PMC5971215

[2] Spence, C. Multisensory contributions to affective touch. *Curr. Opin. Behav. Sci.* **43**, 40–45 (2022).

[3] Sailer, U. & Leknes, S. Meaning makes touch affective. *Curr. Opin. Behav. Sci.* **44**, 101099 (2022).

[4] Nummenmaa, L. et al. Bodily maps of emotions. *Proc. Natl. Acad. Sci. U S A*. **111**, 646–651 (2014). 24379370 10.1073/pnas.1321664111 PMC3896150

[5] Volynets, S. et al. Bodily maps of emotions are culturally universal. *Emotion* **20**, 1127–1136 (2020). 31259590 10.1037/emo0000624

[6] Longo, M. R. et al. More than skin deep: body representation beyond primary somatosensory cortex. *Neuropsychologia* **48**, 655–668 (2010). 19720070 10.1016/j.neuropsychologia.2009.08.022

[7] Winkielman, P. et al. Embodiment of cognition and emotion. American Psychological Association. In *APA handbook of personality and social psychology, Volume 1: Attitudes and social cognition* 1, 151–175 (2015).

[8] Barrett, L. F. & Lindquist, K. A. The embodiment of emotion. Cambridge University Press in Embodied Grounding (eds. Semin, G. R. & Smith, E. R.) 237–262 (2008).

[9] Daikoku, T. et al. Mapping emotional feeling in the body: A tripartite framework for understanding the embodied mind. *Neurosci. Biobehav Rev.* **180**, 106469 (2026). 41207576 10.1016/j.neubiorev.2025.106469

[10] Putkinen, V. et al. Bodily maps of musical sensations across cultures. *Proc. Natl. Acad. Sci. U. S. A.* 121, e2308859121 (2024). 10.1073/pnas.2308859121 PMC10835118 38271338

[11] Ojala, J. et al. Bodily maps of emotions and pain: tactile and hedonic sensitivity in healthy controls and patients experiencing chronic pain. *Pain* **164**, 2665–2674 (2023). 37678245 10.1097/j.pain.0000000000003027 PMC10652713

[12] Walker, S. C. et al. Vicarious ratings of social touch reflect the anatomical distribution & velocity tuning of C-tactile afferents: A hedonic homunculus? *Behav. Brain Res.* **320**, 91–96 (2017). 27915070 10.1016/j.bbr.2016.11.046

[13] Lisi, M. P. et al. Visual perspective and body ownership modulate vicarious pain and touch: A systematic review. *Psychon Bull. Rev.* **31**, 1954–1980 (2024). 38429591 10.3758/s13423-024-02477-5 PMC11543731

[14] Hedger, N. et al. *Nature* 1–9 (2025).

[15] McGlone, F. et al. Discriminative and affective touch: sensing and feeling. *Neuron* **82**, 737–755 (2014). 24853935 10.1016/j.neuron.2014.05.001

[16] Saanijoki, T. & Nummenmaa, L. Bodily maps of exercise-induced feelings. *Sci. Rep.* **15**, 23331 (2025). 40603591 10.1038/s41598-025-07246-5 PMC12223226

[17] Benedetti, F. The sensory and affective components of pain. *Behav. Brain Sci.* **20**, 439–440 (1997).

[18] Price, D. D. Psychological and neural mechanisms of the affective dimension of pain. *Science* **288**, 1769–1772 (2000). 10846154 10.1126/science.288.5472.1769

[19] Lotze, M. & Moseley, G. L. Role of distorted body image in pain. *Curr. Rheumatol. Rep.* **9**, 488–496 (2007). 18177603 10.1007/s11926-007-0079-x

[20] Suvilehto, J. T. et al. Topography of social touching depends on emotional bonds between humans. *Proc. Natl. Acad. Sci. U S A*. **112**, 13811–13816 (2015). 26504228 10.1073/pnas.1519231112 PMC4653180

[21] Bellard, A. et al. Topography and relationship-specific social touching in individuals displaying body image disturbances. *Sci. Rep.* **13**, 13198 (2023). 37580362 10.1038/s41598-023-39484-w PMC10425375

[22] Sailer, U. et al. Disentangling touch aversion: A systematic investigation of measurement approaches, behavioural correlates, and underlying individual differences. *Res. Square*. 10.21203/rs.3.rs-8539862/v1 (2026).

[23] Sailer, U. et al. *Cogn. Emot.* **59**, 565–586 (2024). 10.1080/02699931.2024.2311800 38362744

[24] Hartmann, M. et al. Happiness feels light and sadness feels heavy: introducing valence-related bodily sensation maps of emotions. *Psychol. Res.* **87**, 59–83 (2023). 35226152 10.1007/s00426-022-01661-3 PMC9873729

[25] Harjunen, V. J. et al. Individual differences in affective touch: Behavioral inhibition and gender define how an interpersonal touch is perceived. *Pers. Individ Dif*. **107**, 88–95 (2017).

[26] Jakubiak, B. K. et al. Individual and relational differences in desire for touch in romantic relationships. *J. Soc. Pers. Relat.* **38**, 2029–2052 (2021).

[27] Krahé, C. et al. The meaning of touch: Relational and individual variables shape emotions and intentions associated with imagined social touch. *Eur. J. Soc. Psychol.* **54**, 1247–1265 (2024). 10.1002/ejsp.3076 PMC7616566 39404689

[28] Mehling, W. E. et al. The multidimensional assessment of interoceptive awareness, version 2 (MAIA-2). *PLoS One*. **7**, e48230 (2012). 30513087 10.1371/journal.pone.0208034 PMC6279042

[29] Mariani Wigley, I. L. C. et al. Stroking in early mother-infant exchanges: The role of maternal tactile biography and interoceptive sensibility. *PLoS One*. **19**, e0298733 (2024). 38451923 10.1371/journal.pone.0298733 PMC10919687

[30] Butti, N. et al. To touch or to be touched? comparing appraisal of vicarious execution and reception of interpersonal touch. *PLoS One*. **19**, e0293164 (2024). 38758835 10.1371/journal.pone.0293164 PMC11101113

[31] Wilhelm, F. H. et al. Social anxiety and response to touch: incongruence between self-evaluative and physiological reactions. *Biol. Psychol.* **58**, 181–202 (2001). 11698114 10.1016/s0301-0511(01)00113-2

[32] Ackerley, R. et al. Quantifying the sensory and emotional perception of touch: differences between glabrous and hairy skin. *Front. Behav. Neurosci.* **8**, 34 (2014). 24574985 10.3389/fnbeh.2014.00034 PMC3920190

[33] Ackerley, R. C-tactile (CT) afferents: evidence of their function from microneurography studies in humans. *Curr. Opin. Behav. Sci.* **43**, 95–100 (2022).

[34] Crucianelli, L. et al. Modeling affective touch pleasantness across skin types at the individual level reveals a reliable and stable basic function. *J. Neurophysiol.* **128**, 1435–1452 (2022). 36260710 10.1152/jn.00179.2022

[35] Lee Masson, H. et al. Intact neural representations of affective meaning of touch but lack of embodied resonance in autism: a multi-voxel pattern analysis study. *Mol. Autism*. **10**, 1–14 (2019). 31798816 10.1186/s13229-019-0294-0 PMC6881998

[36] Russell, J. A. A circumplex model of affect. *J. Pers. Soc. Psychol.* **39**, 1161–1178 (1980).

[37] Burleson, M. H. & Quigley, K. S. Social interoception and social allostasis through touch: legacy of the somatovisceral afference model of emotion. *Soc. Neurosci.* **16**, 92–102 (2021). 31810428 10.1080/17470919.2019.1702095 PMC7299836

[38] Fotopoulou, A. et al. Affective regulation through touch: homeostatic and allostatic mechanisms. *Curr. Opin. Behav. Sci.* **43**, 80–87 (2022). 34841013 10.1016/j.cobeha.2021.08.008 PMC7612031

[39] Walker, S. C. et al. Psychophysiology and motivated emotion: testing the affective touch hypothesis of C-tactile afferent function. *Curr. Opin. Behav. Sci.* **43**, 131–137 (2022).

[40] Johansson, C. Views on and perceptions of experiences of touch avoidance: An exploratory study. *Curr. Psychol.* **32**, 44–59 (2013).

[41] Voos, A. C. et al. Autistic traits are associated with diminished neural response to affective touch. *Soc. Cogn. Affect. Neurosci.* **8**, 378–386 (2013). 22267520 10.1093/scan/nss009 PMC3624948

[42] Mello, M. et al. Autism spectrum disorder: The cerebellum, genes, and pathways. *J. Autism Dev. Disord*. **173**, 1–17 (2025).

[43] Boehme, R. et al. Microbiota from young mice counteracts selective age-associated behavioral deficits. *Brain Sci.* **10**, 306 (2020). 37117767 10.1038/s43587-021-00093-9

[44] Lewis, J. S. & Schweinhardt, P. Perceptions of the painful body: the relationship between body perception disturbance, pain and tactile discrimination in complex regional pain syndrome. *Eur. J. Pain*. **16**, 1320–1330 (2012). 22407949 10.1002/j.1532-2149.2012.00120.x

[45] Clauw, D. J. Why don’t we use a body map in every chronic pain patient yet? *Pain* **165**, 1660–1661 (2024). 10.1097/j.pain.0000000000003184 38358934

[46] Mehling, W. E. et al. *PLoS One* **13**, e0208034 (2018). 30513087 10.1371/journal.pone.0208034 PMC6279042

[47] Lee Masson, H. & de Op, H. Intact neural representations of affective meaning of touch but lack of embodied resonance in autism: a multi-voxel pattern analysis study. *PLoS One*. **13**, e0190921 (2018). 31798816 10.1186/s13229-019-0294-0 PMC6881998

[48] Bradley, M. M. & Lang, P. J. Measuring emotion: the self-assessment manikin and the semantic differential. *J. Behav. Ther. Exp. Psychiatry*. **25**, 49–59 (1994). 7962581 10.1016/0005-7916(94)90063-9

[49] Anwyl-Irvine, A. L. et al. *PsyArXiv* doi:10.31234/osf.io/kf5nu (2018).

[50] Available at. (2025). https://www.R-project.org/. (Accessed: 9th December)

