# Supplementary material for: Observing positive and negative social touch triggers topographically distinct physical and emotional reactions in the body
Source: Sci Rep. 2026 Aug 1;16:23721. doi: 10.1038/s41598-026-64569-7 (PMC13428749; doi:10.1038/s41598-026-64569-7)
Supplement: Supplementary file 1 — Supplementary Material [file 41598_2026_64569_MOESM1_ESM.pdf]

Supplementary Material

Observing positive and negative social touch triggers topographically distinct physical and emotional reactions in the body

Justyna Świdrak[1,2], Wenhan Sun[3], Pinar Ekin[3], Merle T. Fairhurst[3]*

Sample details

Seventy-seven participants were European (including 18 British, 17 Polish, and 15 Portuguese), 19 North/South American, and 15 African. The majority of participants had a university degree (N=99, including 45 women); 16 were students. Four participants were unemployed; the rest had some type of employment or owned their own business. Among participants who reported musculoskeletal pain, the most common was back/shoulder pain (N=9), followed by the leg/knee pain (N=5), and fibromyalgia (N=2), with seven participants reporting more than one type of pain.

Plots

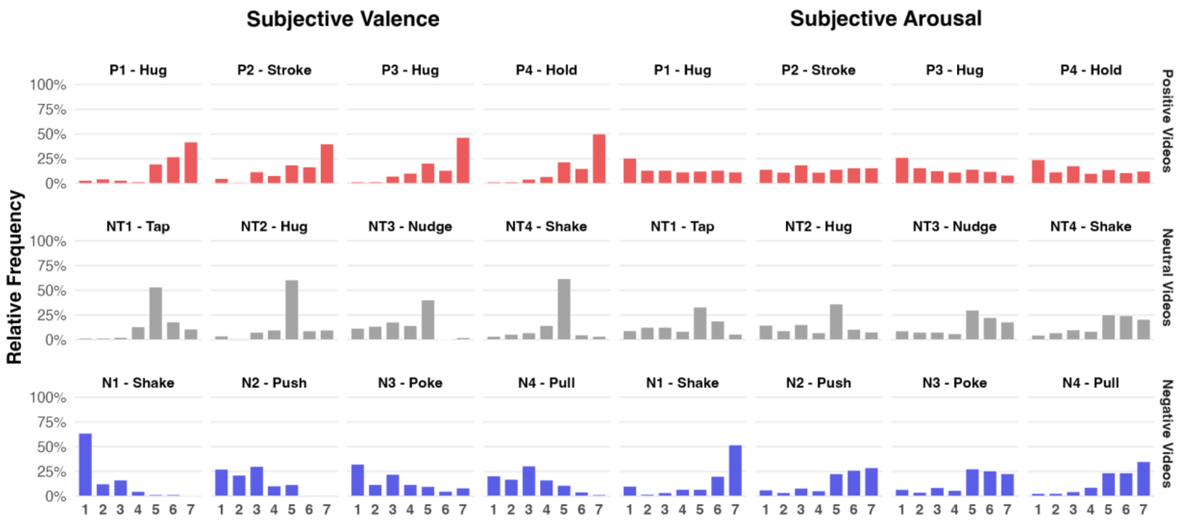

Figure S1. *Raw distribution of subjective arousal (top) and subjective valence (bottom) for positive (, top, from P1 to P4), neutral (middle, from NT 1 to NTl 4) to negative videos (bottom, from N1 to N4). Relative frequencies of participants' ratings on 7-point Likert scales are presented for subjective valence (left) and subjective arousal (right).*

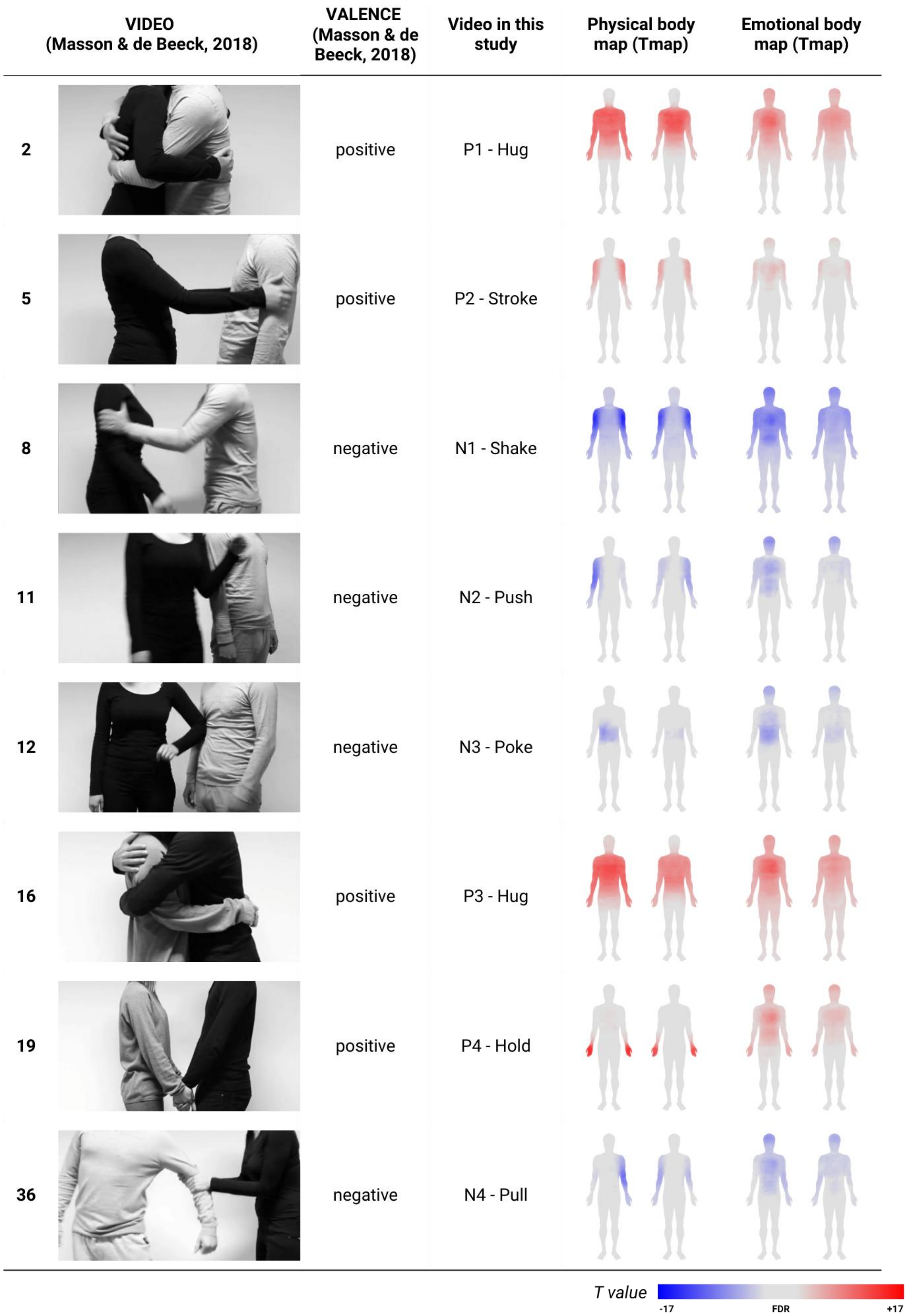

Figure S2. *T-maps for physical and emotional body maps for the positive and negative video stimuli.*

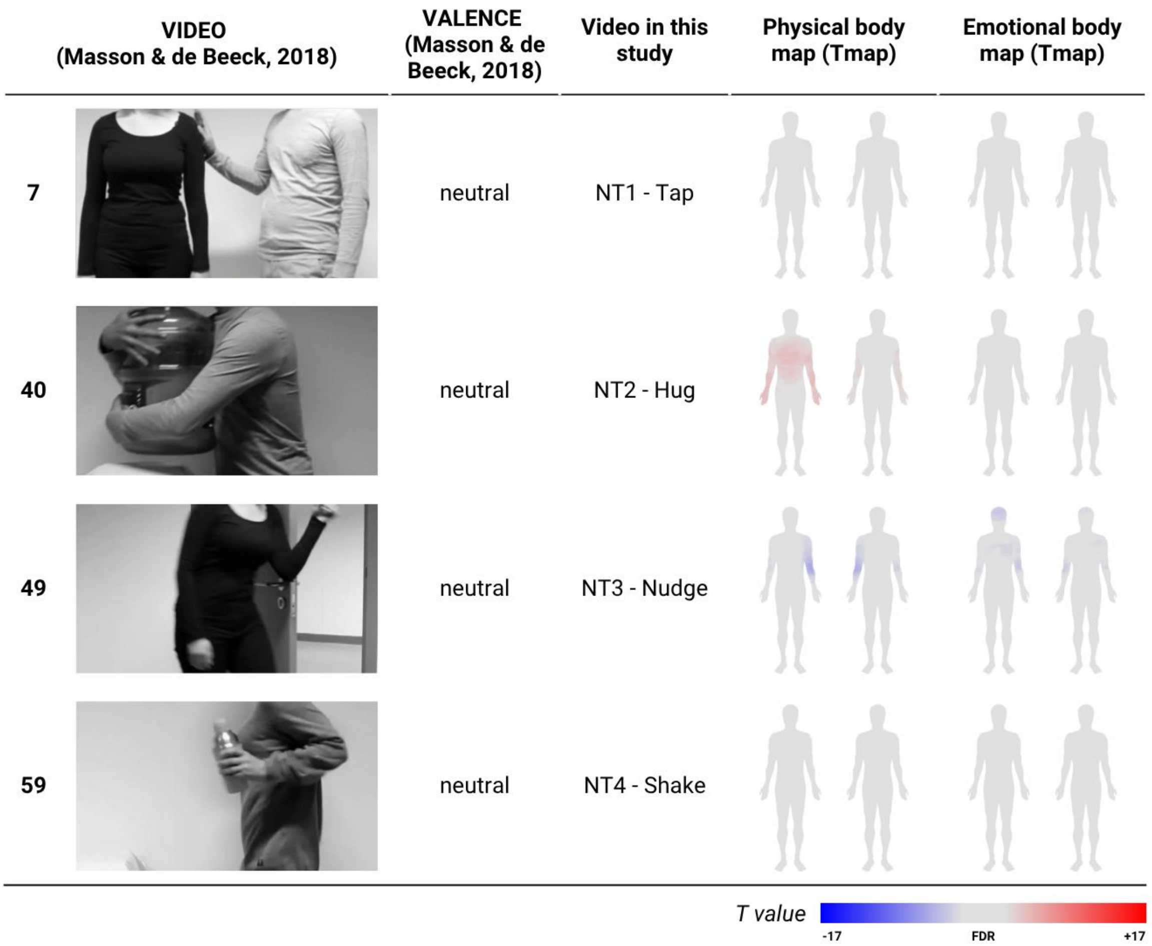

Figure S3. *T-maps for physical and emotional body maps for the neutral (control) stimuli.*

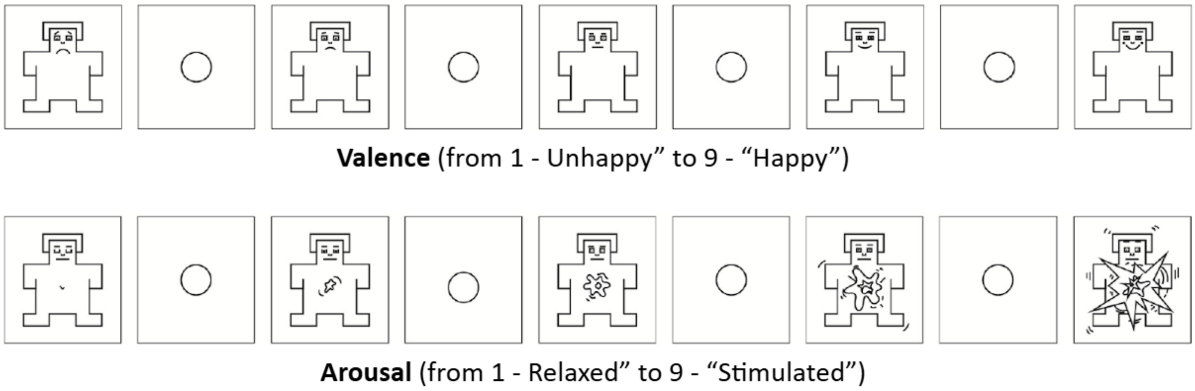

Figure S4. *Self-Assessment Manikins (Bradley and Lang, 1994).*

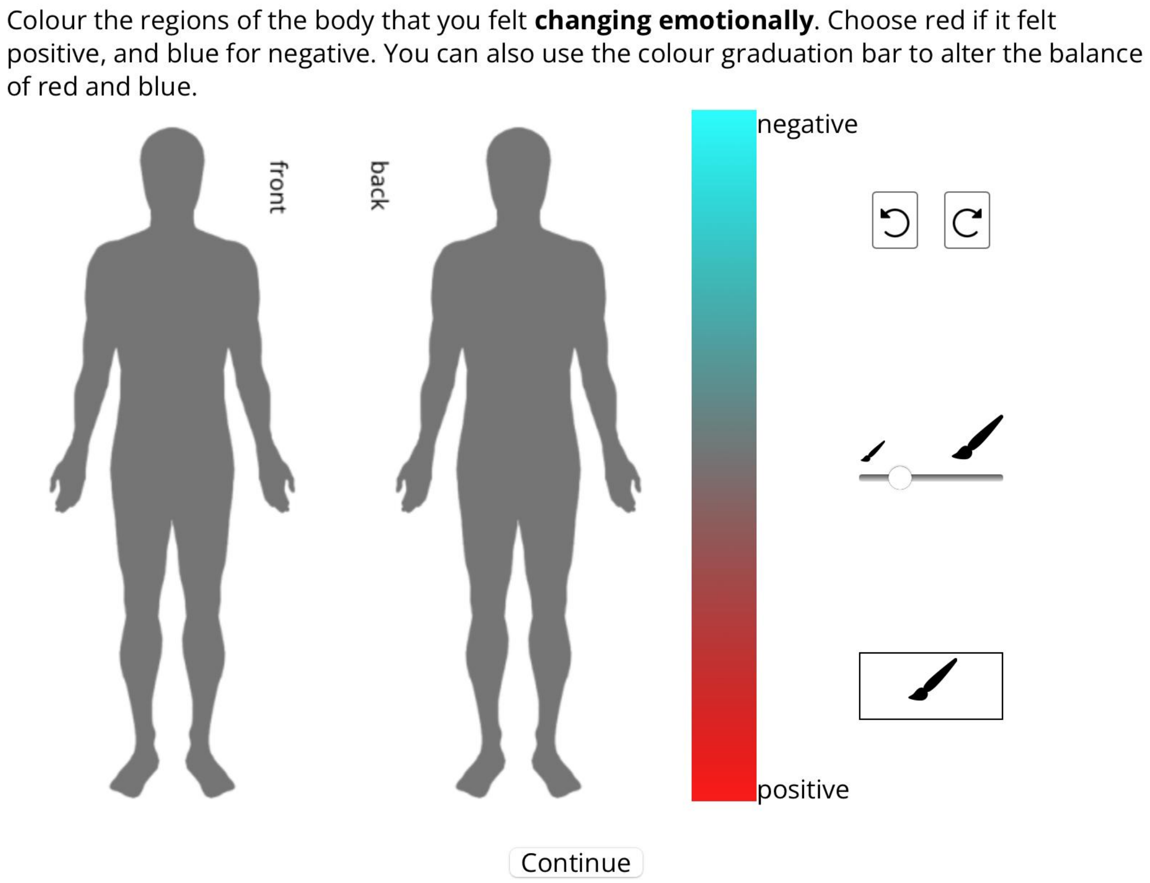

Figure S5. *The BodyMap tool (created by the authors).*

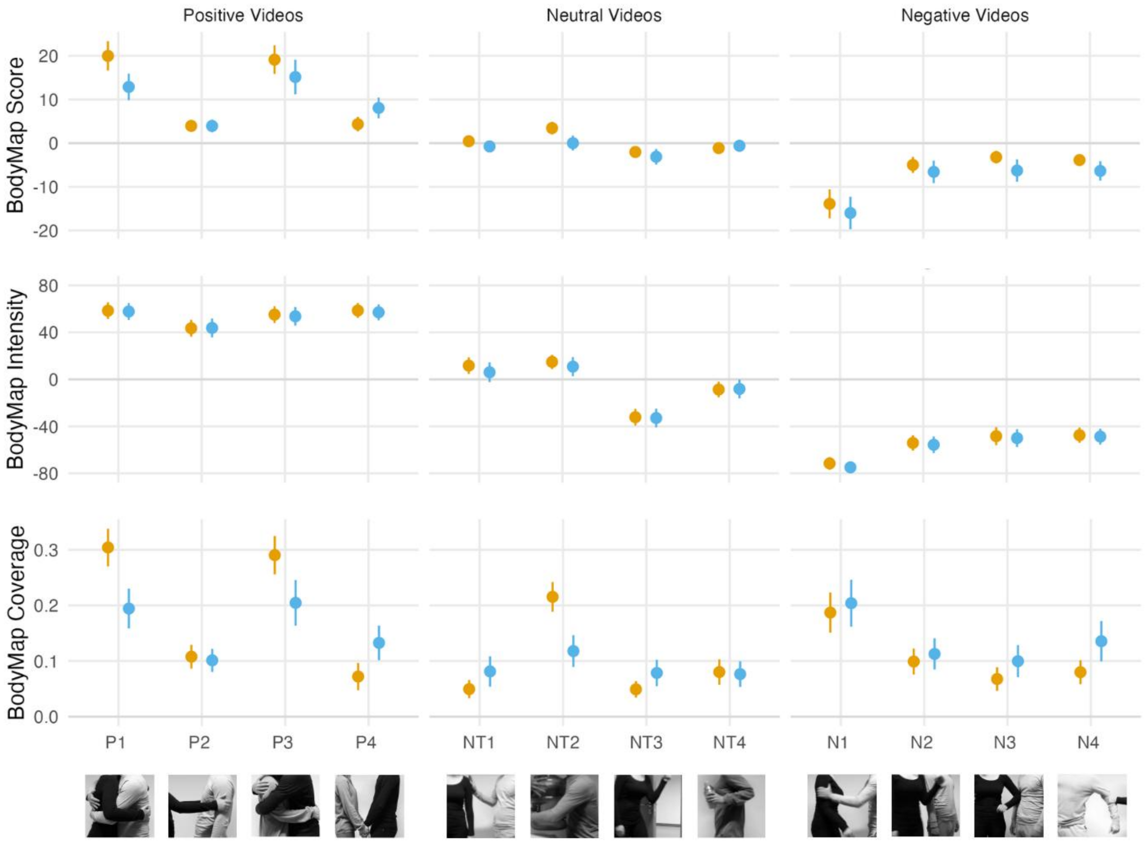

Figure S6. *BodyMap measures (BodyMap Score, BodyMap Intensity, and BodyMap Coverage) for physical and emotional body maps for each positive, neutral, and negative video. Points indicate group means and error bars represent 95% confidence intervals..*

Tables

Table S1. Mean and SD values for BodyMap Score, BodyMap Intensity, BodyMap Coverage for physical and emotional body maps by video.

	BodyMap Score			
	Physical		Emotional	
	Mean	SD	Mean	SD
P1 - Hug	19.979	20.490	12.879	18.719
P2 - Stroke	3.947	8.482	3.941	8.802
P3 - Hug	19.127	19.923	15.137	24.311
P4 - Hold	4.346	10.159	8.061	14.572
N1 - Shake	-13.894	20.469	-15.996	22.792
N2 - Push	-4.989	11.234	-6.570	15.425
N3 - Poke	-3.204	8.827	-6.276	15.386
N4 - Pull	-3.875	7.498	-6.368	13.309
NT1 - Tap	0.427	4.639	-0.747	7.159
NT2 - Hug	3.451	8.735	0.013	9.650
NT3 - Nudge	-2.022	6.700	-3.118	10.120
NT4 - Shake	-1.143	6.797	-0.606	6.152
	BodyMap Intensity			
	Physical		Emotional	
	Mean	SD	Mean	SD
P1 - Hug	58.471	42.364	57.783	43.328
P2 - Stroke	43.499	43.777	43.756	46.463
P3 - Hug	55.147	42.857	53.640	47.110
P4 - Hold	58.629	37.316	57.055	40.006
N1 - Shake	-71.552	32.355	-74.893	27.805
N2 - Push	-54.095	38.777	-55.672	41.661
N3 - Poke	-48.357	45.597	-49.973	44.324
N4 - Pull	-47.500	38.964	-48.722	39.457
NT1 - Tap	11.697	42.295	6.035	47.393
NT2 - Hug	14.877	36.319	10.890	43.548
NT3 - Nudge	-32.192	41.604	-32.905	44.275
NT4 - Shake	-8.631	40.118	-8.146	43.474

	Coverage

	Physical		Emotional	
	Mean	SD	Mean	SD
P1 - Hug	0.304	0.207	0.194	0.220
P2 - Stroke	0.108	0.132	0.101	0.125
P3 - Hug	0.290	0.211	0.205	0.251
P4 - Hold	0.072	0.151	0.133	0.190
N1 - Shake	0.187	0.222	0.204	0.257
N2 - Push	0.099	0.142	0.113	0.168
N3 - Poke	0.068	0.131	0.100	0.175
N4 - Pull	0.080	0.132	0.136	0.217
NT1 - Tap	0.049	0.099	0.081	0.159
NT2 - Hug	0.215	0.161	0.118	0.159
NT3 - Nudge	0.049	0.089	0.079	0.134
NT4 - Shake	0.080	0.138	0.076	0.130

Table S2. Mean and SD values for subjective ratings of valence and arousal for each video.

	Subjective Valence		Subjective Arousal	
	Mean	SD	Mean	SD
P1 - Hug	7.331	1.793	4.453	2.716
P2 - Stroke	6.378	2.062	4.473	2.299
P3 - Hug	7.257	1.811	3.899	2.476
P4 - Hold	7.270	1.717	4.203	2.545
N1 - Shake	1.750	1.200	7.480	1.991
N2 - Push	2.615	1.373	6.007	2.062
N3 - Poke	3.095	2.008	6.041	2.102
N4 - Pull	2.980	1.473	6.196	1.772
NT1 - Tap	5.318	1.310	4.365	1.867
NT2 - Hug	5.223	1.560	4.149	1.939
NT3 - Nudge	3.709	1.549	5.135	2.056
NT4 - Shake	4.588	1.277	5.372	1.942

Table S3. Results from mixed effect linear regression, predicting body map score with body map type, video valence, with random intercepts for participant and video.

lmer: body map score ~ body map type + video valence + (1|ID) + (1|video)						
	Estimate	Std. Error	df	t value	Pr(>|t|)	
(Intercept)	-6.614	3.003	6.242	-2.202	0.068	.
body map type - emotional	-2.087	0.657	2142.486	-3.178	0.002	**
video valence - positive	18.567	4.205	6.002	4.415	0.004	**

Conditional R2:	0.349					
Marginal R2:	0.230					

Table S4. Results from mixed effect linear regression, predicting body map score with body map type, and subjective ratings of valence and arousal, with random intercepts for participant and video.

lmer: body map score ~ body map type + subjective valence + subjective arousal + (1|ID) + (1|video)						
	Estimate	Std. Error	df	t value	Pr(>|t|)	
(Intercept)	-5.730	2.279	17.997	-2.514	0.022	*
body map type - emotional	-1.976	0.627	2135.126	-3.153	0.002	**
subjective valence	2.669	0.195	1065.576	13.715	< 2e-16	***
subjective arousal	-0.837	0.150	1688.486	-5.588	0.000	***

Conditional R2:	0.329					
Marginal R2:	0.239					

Table S5. Results from linear mixed-effects regression predicting body map scores from body map type, video valence, touch aversion (STQ), MAIA score, pain status, and gender, including interactions between video valence and STQ, MAIA score, and pain status, with random intercepts for participants and videos.

lmer: bodymap score ~ body map type + video valence * touch aversion + valence * MAIA + pain status+ gender + (1 | ID) + (1 | video)						
	Estimate	Std. Error	df	t value	Pr(>|t|)	
(Intercept)	-8.052	4.388	26.715	-1.835	0.078	.
bodymap type - emotional	-2.055	0.666	2094.920	-3.087	0.002	**
video valence - positive	25.745	5.727	19.805	4.495	0.000	***
touch aversion (STQ)	0.041	0.045	364.524	0.905	0.366	
MAIA	-0.153	0.826	362.152	-0.186	0.853	
pain - yes or unsure	-3.688	1.265	361.020	-2.915	0.004	**
gender - male	1.882	0.845	137.390	2.226	0.028	*
video valence - positive : touch aversion	-0.212	0.056	2087.301	-3.768	0.000	***
video valence - positive : MAIA	0.152	1.027	2095.062	0.148	0.882	
video valence - positive : pain - yes or unsure	3.116	1.576	2089.693	1.976	0.048	*

Conditional R2:	0.356					
Marginal R2:	0.242					
